# Working from home and management controls

**DOI:** 10.1007/s11573-022-01123-7

**Published:** 2022-12-13

**Authors:** Konstantin Flassak, Julia Haag, Christian Hofmann, Christopher Lechner, Nina Schwaiger, Rafael Zacherl

**Affiliations:** 1 Institute for Accounting and Control, LMU Munich School of Management, Ludwigstraße 28, 80539 Munich, Germany; 2 Robert Bosch GmbH, Robert-Bosch-Platz 1, 70839 Gerlingen-Schillerhöhe, Germany; 3 Oliver Wyman GmbH, Müllerstraße 3, 80469 Munich, Germany

**Keywords:** Covid-19 pandemic, Working from home, Management control, Employee outcomes, M12, M41, M54

## Abstract

The Covid-19 pandemic and the corresponding shift toward working from home (WFH) amplifies control problems within organizations and poses severe challenges for management control as employees’ tasks are difficult to observe under WFH conditions. We examine the association between WFH and action controls. Based on a survey among employees in a large international corporation, we find that under WFH conditions the organization more intensively uses standardization and planning participation. We also examine the association between WFH and employee outcomes. The findings suggest that WFH is associated with more time employees spend in meetings and a higher job focus. Overall, the study adds to the literature by exploring the association between WFH and the use of management controls in organizations.

## Introduction

Due to the Covid-19 pandemic, many organizations converted to working from home (WFH) arrangements to comply with regulatory requirements, protect their employees’ health, and remain productive (e.g., Alipour et al. 2020). Barrero et al. (2021), among others, find that WFH is associated with positive consequences for employees, employers, and society at large, suggesting that WFH is likely to continue in the future. However, the shift to WFH poses severe challenges for organizations’ management control (MC). In particular, WFH likely reduces the effectiveness of MCs to steer employees toward taking actions that are consistent with organizational objectives. For example, under WFH conditions, employee actions are less observable, and the use of direct monitoring is limited. We analyze how the Covid-19 pandemic and the corresponding shift toward WFH shapes the design of MCs and, in turn, explore the role of MCs in explaining the association between WFH and employee outcomes such as working day structure and job focus.

Literature in psychology and management documents the benefits and costs associated with WFH. For instance, Gajendran and Harrison (2007) and Martin and MacDonnell (2012) suggest that WFH is associated with an increase in job satisfaction and organizational commitment. De Menezes and Kelliher (2011) and Bloom et al. (2015) show that WFH is associated with higher organizational performance. In contrast, WFH intermingles employees’ work-life and private-life, potentially resulting in mental problems such as stress (Alipour et al. 2020). Due to the physical separation from the workplace, the supervisor, and the colleagues, WFH may foster employees’ professional isolation (Golden et al. 2008).

Our study examines the design of MCs and the associated employee outcomes under WFH conditions. The Covid-19 pandemic and the corresponding shift toward WFH establish physically dispersed working environments. Such working environments amplify control problems within organizations and thus pose several challenges to the design of MCs. First, under WFH conditions, employees’ actions are less observable, limiting the possibilities for direct monitoring (Greer and Payne 2014; Allen et al. 2015) and potentially increasing the likelihood of mistakes and shirking. Second, WFH hampers the information exchange between employees and their supervisors as well as among employees. While the former reduces a supervisor’s effectiveness in providing direction and support (Bonet and Salvador 2017), the latter leads to less and more effortful communication (Gajendran and Harrison 2007; Lill 2020). Third, the professional isolation caused by WFH diminishes employee motivation and may lead to adverse job performance and increased turnover (Golden et al. 2008).

Organizations use their MCs to ensure that employees’ behaviors and decisions are consistent with the organization’s strategies and objectives (Anthony and Govindarajan 2004; Merchant and Van der Stede 2017), ultimately attaining fit and enhanced performance (Chenhall 2003). Generally, organizations select from a set of MC practices such as action, result, personnel, and cultural controls (Merchant and Van der Stede 2017). In the main analysis, we investigate how organizations adjust their MCs due to the Covid-19 pandemic and the associated shift toward WFH. During the pandemic, organizations are more likely to adapt *action* controls, as the associated control practices such as standardization, pre-action reviews, and planning participation are more easily adjusted in the short-term. Thus, we expect that organizations strengthen their action controls to mitigate the amplified control problems resulting from WFH conditions.

Relying on data from a survey distributed among 2855 employees without supervisory function in a large international multi-divisional service firm from March until April 2021, we find support for the predictions. First, the firm experienced a significant increase in WFH: On average, WFH has increased from 5 h per week in 2019 to 27 h per week in 2021. Second, we provide evidence that WFH is associated with amplified firm control problems. We find that, under WFH conditions, supervisors lack observability of employees’ actions; socialization processes are less prevalent under WFH conditions, arguably increasing social isolation and thus decreasing organizational identification and employee motivation. Third, we provide evidence of strengthened action controls. We find that supervisors more strongly standardize tasks of employees working from home and that supervisors more strongly involve these employees in their strategic planning. A mechanism analysis suggests that these results are explained by the supervisor’s lack of observability of employees’ actions rather than the hampered communication or socialization under WFH conditions.

In an additional analysis, we examine the association between WFH and employee outcomes (such as meeting hours and employee focus) and the moderating role of MCs. This analysis provides further insights into the benefits and costs of action controls. We document that the number of hours employees spend in meetings is significantly higher in 2021 as compared to 2019, which is partly explained by the strong increase in WFH from 2019 to 2021. Interestingly, this association is only statistically significant in the subsample of weak action controls. This finding suggests that strong action controls reduce the necessity for time-consuming meetings. In addition, we find that employees are better able to focus on their job in 2021 as compared to 2019 when they experienced a strong increase in WFH.

The study contributes to the literature in three ways. First, we contribute to the literature by investigating how WFH is associated with the design of MCs. The Covid-19 pandemic pushed organizations toward WFH. As WFH is likely to stick in the future as a new way of working (Barrero et al. 2021), there is an increased interest in the association between WFH and MCs. We show that WFH amplifies organizations’ control problems of lack of direction, lack of motivation, and personal limitations, suggesting that organizations consider the role of WFH in setting up their MCs.

Second, physically dispersed teams are ubiquitous in modern organizations (Bonet and Salvador 2017). For example, professional service firms or IT companies often employ teams where team members work at different places. Such arrangements pose challenges to collaboration and communication among team members and supervisors (Dulebohn and Hoch 2017). We study how supervisors design their MCs in situations where employees are physically dispersed. We thereby add to the literature investigating organizational structure as an important contingency factor for the effectiveness and design of MCs (e.g., Abernethy et al. 2004; Gerdin 2005; Kristensen and Israelsen 2014).

Third, we examine how WFH in conjunction with MCs is associated with employee outcomes. Prior studies address the consequences of WFH on individual and organizational outcomes such as job satisfaction or turnover (Gajendran and Harrison 2007; De Menezes and Kelliher 2011), productivity (e.g., Bloom et al. 2015), and work patterns (e.g., Gibbs et al. 2022). For example, Bloom et al. (2015) investigate the effects of WFH on productivity, such as calls per minute for employees of a Chinese travel agency. We investigate how the design of MCs moderates the association between WFH and employee outcomes such as meeting hours and employee focus.

The remainder of the paper is organized as follows. Section 2 reviews previous literature and develops the hypothesis. Section 3 presents the empirical study, including data, sample, and variable measurement. Section 4 reports the main findings, the robustness checks, and additional analysis. Section 5 concludes.

## Literature and hypothesis development

### Covid-19 and WFH

The Covid-19 pandemic had severe impacts on the economic activity of firms. In the short-term, the decrease in sales and liquidity led to a strained financial situation of numerous German firms (Bundesbank 2021). For example, for a representative sample of German firms, Bischof et al. (2020) find that approximately 60% of firms experienced a sales decrease of more than 10% due to the pandemic. Besides using governmental aid programs, firms took cost-cutting measures, limited their cash outflows, and increased their demand for loans (Bischof et al. 2022; Kfw 2021). Bloom et al. (2021) document a significant drop in business due to the Covid-19 pandemic for a broad sample of US firms.

Besides, governments all around the globe imposed non-pharmaceutical interventions such as obligatory WFH to reduce the spread of the virus. Due to the pandemic, WFH has increased substantially. In Germany, 34% of employees worked partly or entirely from home in April 2020 compared to only 12% of employees before the pandemic (Schröder et al. 2020; DIW Berlin 2016). In the US, the percentage of paid full days worked from home rose from five percent to over 60 percent in May 2020 and remained between 50 and 43 percent in 2021 (Barrero et al. 2021).

Literature documents various consequences of WFH. At the organizational level, WFH enables organizations to attract a larger and more diverse talent pool (Eversole et al. 2012), reduce absenteeism (De Menezes and Kelliher 2011), and lower real estate costs (Perez et al. 2002). However, WFH poses challenges regarding the provision of an effective work environment and in terms of compliance issues related to data protection (McKinsey 2020); and legal issues such as income taxation, the recording of hours worked, and insurances in case of work accidents (Forbes 2021).

At the individual level, positive effects of WFH include increased organizational commitment and job satisfaction (Gajendran and Harrison 2007; Martin and MacDonnell 2012). For example, WFH increases employees’ flexibility when and how work is done. Employees save commuting time and stay closer to their families, which improves work-life balance (Brownson 2004). WFH also allows employees to escape undesirable features of the office workplace, such as noise or interruptions by colleagues (Sewell and Taskin 2015). However, WFH diminishes employees’ clear distinction between work and private-life (Ashforth et al. 2000; Raghuram and Wiesenfeld 2004), potentially causing mental problems like stress (Alipour et al. 2020). Related, the physical separation from the workplace and their co-workers can increase employees’ perceived isolation (Golden et al. 2008). And finally, the missing salience of organizational facilities and symbols likely reduces the employees’ identification with the organization (e.g., Illgems and Verbeke 2004; Sardeshmukh et al. 2012).

Several studies investigate how WFH affects employee productivity. Angelici and Profeta (2020) document in a field experiment that granting employees time and space flexibility increases their productivity. Bloom et al. (2015) conduct a WFH field experiment with employees from a Chinese travel-agency call center; they find that WFH increases productivity by 13%. In contrast, relying on observational data, Gibbs et al. (2022) find that employee productivity fell for a sample of 10,000 Indian IT professionals who shifted abruptly to WFH during the Covid-19 pandemic.

The literature also investigates the changes in work patterns when employees shift to WFH. Teevan et al. (2020) and Yang et al. (2022), using detailed communication data of US Microsoft employees, document an increase in the number of meetings and meeting participants. Employees’ communication under WFH relies on modern telecommunication devices. The devices alter the amount and quality of exchanged information and the way co-workers communicate and coordinate among each other (e.g., Workman 2005; Gajendran and Harrison 2007), potentially resulting in higher communication costs (Gibbs et al. 2022; Yang et al. 2022). At the same time, WFH increases total working hours (DeFilippis et al. 2020) but decreases uninterrupted working hours (Gibbs et al. 2022). What remains open is how the design of MCs moderates the association between WFH and employee outcomes.

### MCs

Management Control (MC) refers to all procedures, methods, and devices to ensure that employees’ behaviors and decisions are consistent with the organization’s strategies and objectives (Anthony and Govindarajan 2004). The set of applied MC practices forms the organization’s MC system. The organization establishes MCs because employees may not act in the best interest of the organization (Jensen and Meckling 1976). According to Merchant and Van der Stede (2017), control problems emerge because employees do not know what the company expects from them (i.e., lack of direction); employees pursue their own interests that do not coincide with the organizational objectives (i.e., lack of motivation); employees do not have the necessary resources and skills to perform the tasks assigned to them (i.e., personal limitations).

In addressing the control problems, the organization chooses from diverse MC practices. Merchant and Van der Stede (2017) classify MC practices according to the object of control. Within this framework, the organization’s MC practices focus on the actions taken by employees (action control), the results produced by employees (result control), the values and norms shared among employees (cultural control), and the type of people employed (personnel controls).

In this study, we focus on the design of action controls under WFH conditions caused by the Covid-19 pandemic. Action controls represent the “most direct form of management control” (Merchant and Van der Stede 2017, p.86) and ensure that employees perform specific actions that contribute to the organization’s success. To that end, action controls restrict employees’ discretion by specifying boundaries to acceptable behavior or by implementing formal approval procedures (Simons 1995). Action controls are effective when the employee knows the actions required to obtain a particular outcome, and control is achieved by monitoring adherence to standardized rules and procedures (Ouchi 1977).

The focus on action controls is motivated by the MCs’ adaptability. The pandemic-related push toward WFH changes organizations’ external environment, requiring an adaptation of organizations’ MCs to attain fit and enhanced performance (Chenhall 2003). However, organizations might not be able to adjust all MC practices in the short-term as an organization’s MC design focuses on efficiency rather than adaptability (Otley 1994). As implementing result (e.g., performance measurement), personnel (e.g., employee selection), and cultural controls (e.g., codes of conduct) requires large organizational effort, these MCs are not likely to be adjusted in the short-term. We argue that such constraints are weaker for action controls.

Specifically, we focus on the action controls: (1) Standardization; (2) Planning participation; and (3) Pre-action reviews. *Standardization* refers to guidelines and procedures that specify the means of the conducted work activities (Daft and Macintosh 1984). Standardization addresses a lack of direction as well as personal limitations because employees can follow guidelines and apply pre-determined procedures. *Planning participation* refers to the involvement of employees in strategic planning processes and the pre-determination of activities. The participation of employees in the planning process aims to achieve goal congruence (Coch and French Jr 1948; Nutt 1989) because employees will be familiar with the organization’s objectives, enabling employees to focus on organizational goals (Ketokivi and Castaner 2004). Thus, planning participation provides direction to employees and addresses motivational concerns. With *pre-action reviews*, supervisors scrutinize their employees’ action plans such that supervisors can approve or disapprove certain actions or ask for modifications and re-adjustments. Pre-action reviews guide or restrict proposed activities (Merchant and Stede, 2017), providing direction and resolving personal limitations.

According to contingency theory (Otley 1980), an organization selects the design of its MCs such that the controls match the internal and external environment. In other words, an organization trades off the benefits and costs of the MC practices in the respective context to implement an effective MC system. Prior work examines the choice and effectiveness of MCs with regard to contingency factors such as strategy (e.g., Bisbe and Otley 2004; Widener 2007; Bedford et al. 2016), organizational structure (e.g., Abernethy et al. 2004; Gerdin 2005; Kristensen and Israelsen 2014), culture (e.g., Harrison and McKinnon 1999; Heinicke et al. 2016; Malmi et al. 2020), and environmental uncertainty (e.g., Grabner et al. 2018; Gerdin et al. 2019). Below, we elaborate on the benefits and costs of MC practices under WFH conditions.

### Hypothesis development

Given an organization’s control problem, the organization trades off the benefit and cost of the MC practices when designing its MC system (Milgrom and Roberts 1992; Roberts 2004). For action controls, the benefit relates to the mitigated control problems. The cost relates to administrative resources (e.g., the salaries of supervisors or employees who set up and enforce action controls) and the investments to install monitoring devices.

We expect that WFH amplifies the organization’s control problem and thus increases the benefit of action controls. Generally, WFH involves a lack of observability, professional isolation, and a hampered information exchange. First, the lack of observability of an employee’s actions limits the supervisor’s possibility for direct monitoring (Felstead et al. 2003; Greer and Payne 2014; Allen et al. 2015), increasing the probability of mistakes and shirking. Related, the physical separation of the employee from their co-workers reduces the scope for beneficial mutual monitoring (Arya et al. 1997), reducing the effectiveness of alternative means to address the control problem. Second, due to the lack of face-to-face interaction and informal exchange with co-workers, the employee feels professionally isolated, out of touch with their peers (Illgems and Verbeke 2004; Lill 2020), and suffers motivational challenges, resulting in increased turnover intention (Golden et al. 2008). Third, the information exchange between the employee and the supervisor is more challenging, reducing the supervisor’s opportunity to provide guidance and mentoring (Bonet and Salvador 2017). The hampered information exchange is potentially mitigated by the use of modern communication technologies such as instant messaging or video communication (Yang et al., 2022).

The cost of implementing action controls such as standardization, planning participation, and pre-action reviews is arguably similar under WFH conditions compared to an office working environment. Thus, we expect that the organization strengthens the use of action controls under WFH conditions.^1^ Consequently, we state:

**H1:** Action controls are stronger when employees work from home.

## Empirical study

### Sample and data collection

We test the prediction using a survey sent to employees of a large international multi-divisional service firm. The research site represents a promising setting for two reasons: First, the firm’s divisions offer a large and diverse spectrum of services, which requires different MCs. Thus, the research site allows us to examine the use and effectiveness of MC practices for a broad range of business types, even though the study is conducted within a single firm. Second, the firm’s divisions differ in the extent to which they enable the use of WFH (for example, due to data security restrictions or the extent of on-site customer services). Thus, employees face variations in the strength of the *WFH shock* due to the Covid-19 pandemic (i.e., the increase in WFH hours per week).^2^ Interviews with the firm's management revealed that, broadly stated, the firm’s businesses were only mildly affected by the pandemic. That is, neither sales declined substantially nor was there a significant change in the employees’ tasks due to the pandemic.

To inform the development of the survey, we first conducted interviews with the head of management accounting to gather a general understanding of the MCs employees are exposed to in their daily work. We designed the survey to reflect the insights from the interview. The survey consists of four parts: Questions on (1) the prevalence of WFH, (2) the use of MC practices according to the framework by Merchant and Stede (2017), (3) employee outcomes, and (4) control variables. Questions cover two periods: Before and after the first Covid-19 lockdown period (i.e., in 2019 and 2021, respectively).^3^

We distributed the questionnaire via an online survey in March and April 2021 among 2855 employees without supervisory function in seven divisions of the firm and reached a response rate of roughly 60%. To guarantee anonymity, we used the custodian approach (Vogel 2018), i.e., one employee from the HR department of each division distributed the survey to the respondents. After cleaning the dataset, the final sample contains 2576 employee-year observations for the years 2019 and 2021. Table 1 presents the sample selection procedure.

**Table 1:** Sample Selection

Steps	Number	Proportion
Recipients	2855	
Participants	1707	60%
Employee-year observations (years 2019 and 2021)	3414	
Sample derivation		
Less observations with missings in WFH data	3250	
Less observations with missings in action control data	2589	
Less observations with missings in control variable data	2576	
Final sample	2576	

Since we collect the variables from a survey, the results could be exposed to the common method bias. To reduce this concern, we took established procedural steps ex-ante, such as avoiding ambiguity in the survey questions, guaranteeing employees’ anonymity by the custodian approach, and providing respondents with a cover story that separates the key variables. Furthermore, we conduct a Harman’s one factor test by applying an unrotated principal components analysis to assess common method bias (Bedford and Malmi 2015; Chin 2010). The first factor explains 30.6% of the variance, indicating no evidence for the presence of a common method bias (Podsakoff and Organ 1986).

To reduce the concern of a non-response bias, we provided respondents with a comfortable survey setting by allowing them to interrupt the survey at any time and continue later. Additionally, we promoted survey participation via the custodians. To test whether the results are subject to a non-response bias, despite the implemented measures, we rely on the extrapolations approach suggested by Armstrong and Overton (1977) and compare observations from early and late responders using univariate ANOVAs. We find no statistically significant differences (i.e., *p* > 0.10) in the main dependent and independent variables when comparing data of respondents who completed the survey before and after we have sent out a reminder (i.e., April 13th 2021).

### Regression model

To examine Hypothesis H1 that WFH is associated with stronger action controls, we run the following regression model:

1 $${\mathbf{MC}}_{it} = \beta_{0} + \gamma WFH_{it} + {\mathbf{\theta Tenure}}_{i} + \tau Teamsize_{i} + \eta_{k} + \varepsilon_{it} ,$$

where *i* captures the employee, *t* reflects the year, and *k* reflects the employee’s division of the firm. ${\mathbf{MC}}$ is a vector capturing aggregate action control and the single action control practices standardization, planning participation, and pre-action reviews. $WFH$ captures the employee’s exposure to WFH. ${\mathbf{Tenure}}$ is a vector capturing three types of employee tenure measures. $Teamsize$ is the size of the team or department the employee is working in. $\eta_{k}$ captures division fixed effects. The regression model is estimated by ordinary least squares (OLS) with robust standard errors clustered at the employee level. Following Hypothesis H1, we expect $\gamma$ to be positive for each measure of action control, suggesting that WFH is associated with stronger action controls.

### Variable measurement

We collected the main variables through reflective and formative multi-item constructs measured on a 7-point Likert scale: 1 for “very small extent” and 7 for “very high extent.” Wherever possible, we rely on pre-established measures from literature. When there is no appropriate construct available in the literature, we suggest a corresponding measure while relying on similar established constructs and definitions from previous work.

Since the research site predominantly employs native German employees, we translated the survey into German to address potential language barriers. To guarantee validity, we asked an academic who is not affiliated to this study to back-translate the survey into English. Before distributing the survey, we collected feedback from academics as well as practitioners. Based on this feedback, we conducted minor verbal adjustments to make sure that the respondents understand the survey questions.

#### Main variables

We capture WFH by the number of hours employees work from home. In particular, *WFH* is the natural logarithm of the number of hours worked from home per week, as indicated by the respondent for the years 2019 and 2021. We acknowledge that *WFH* reflects the realized WFH time and not necessarily the intended WFH time.

We capture action controls in three different types: (1) standardization, (2) planning participation, and (3) pre-action reviews. The constructs are based on Bedford and Malmi (2015). *Standard* is a three-item reflective construct, capturing to what extent there are rules and procedures in place that specify how work activities have to be conducted. *PlanPart* captures to what extent employees are involved in strategic planning processes. *ActRev* is a two-item formative construct, capturing to what extent there are processes of scrutinization and authorization before an activity is performed by an employee. The final variables represent the latent variable scores obtained from construct validation of all items related to the single constructs.^4^

We assume that the supervisor’s use of action controls (*ActCtrl*) is a higher-order formative construct that is an aggregate of the three lower-order constructs *Standard*, *PlanPart*, and *ActRev*. We determine *ActCtrl* by taking the latent variable scores obtained from construct validation of the constructs *Standard*, *PlanPart*, and *ActRev*. We report the survey questions and response scales in Appendix 1 and perform construct validity analyses in Appendix 2.^5^

#### Control variables

We capture the size of the control problem by three variables reflecting task programmability, ease of communication, and socialization. Task programmability (*TaskProg*) is the extent that supervisors are able to observe employees’ actions and to anticipate action-outcome relations. *TaskProg* is measured as a three-item reflective construct used by Bedford and Malmi (2015). The final variable is the latent variable score obtained from construct validation of all items related to task programmability in the respective year as indicated by the respondent. Communication (*Comm*) accounts for the difficulty of the information exchange. *Comm* is a score capturing the extent to which informal communication is used in passing information up and down the hierarchy in the respective year as indicated by the respondent. The variable *Social* is measured by a three-item formative construct based on Bedford and Malmi (2015) and captures to what extent there are processes in place that emphasize organizational values and norms. The final variable is the latent variable score obtained from construct validation of all items related to socialization in the respective year indicated by the respondent.

We control for different types of employee tenure to account for potential differences in the use of MCs related to employee experience or the trust between supervisors and employees. *TenureBoss* captures the natural logarithm of the midpoint of the range indicated as tenure with the current supervisor in years. *TenurePos* captures the natural logarithm of the midpoint of the range indicated as tenure in the current position in years. *TenureFirm* captures the natural logarithm of the midpoint of the range indicated as tenure with the company in years.

We also control for the size of the team the employee is working in, which likely explains the effectiveness and thus use of MCs. *Teamsize* is measured as the natural logarithm of the total number of employees working in the same team or department. Finally, we add division fixed effects to account for potential differences in the applicability of MCs across the divisions at the research site.

## Results

In this section, we present the regression results of the study. In Sect. 4.1, we report on the descriptive statistics. In Sect. 4.2, we elaborate upon the main findings related to Hypothesis H1, stating that WFH is associated with stronger action controls. In Sect. 4.3, we report on the mechanism driving Hypothesis H1. In Sect. 4.4, we report on an additional analysis that emphasizes the implications of the main findings. More specifically, we study how the shift toward WFH is associated with employee outcomes, such as employees’ working day structure and the extent to which employees focus on their job, and how the organization’s MC choice moderates the association between WFH and employee outcomes.

### Descriptive statistics

Table 2 presents the descriptive statistics on the variables. In Panel A, we report on the full sample. In Panel B, we report on the change in the control problem from before (i.e., 2019) to during the pandemic (i.e., 2021). In Panel C, we compare the main variables for low and high WFH (i.e., we split the sample at the median of *WFH*).

**Table 2:** Descriptive Statistics

Panel A: Full sample					
Variable	Mean	Std. Dev	Min	Median	Max
WFH^a^	15.93	15.99	0.00	8.00	50.00
ActCtrl^b^	3.86	1.12	1.00	3.83	7.00
Standard^b^	4.42	1.33	1.00	4.67	7.00
PlanPart^b^	3.24	1.90	1.00	3.00	7.00
ActRev^b^	3.91	1.57	1.00	4.00	7.00
TenureBoss^a^	3.61	3.09	1.00	1.50	10.00
TenurePos^a^	5.59	3.60	1.00	5.00	10.00
TenureFirm^a^	6.93	3.51	1.00	8.00	10.00
Teamsize^a^	14.87	17.57	2.00	10.00	202.00
TaskProg^b^	4.63	1.47	1.00	4.67	7.00
Comm	4.39	1.52	1.00	4.00	7.00
Social^b^	3.88	1.51	1.00	4.00	7.00

Panel B: Change in the control problem from before to during the pandemic			
Variable	2019	2021	Diff./sign
WFH^a^	4.75	27.02	22.27***
TaskProg^b^	4.68	4.57	− 0.11*
Comm	4.44	4.34	– 0.10
Social^b^	4.42	3.35	− 1.07***

Panel C: Comparison of action controls for low and high WFH			
Variable	Low WFH	High WFH	Diff./sign
ActCtrl^b^	3.80	3.91	0.12***
Standard^b^	4.33	4.50	0.17***
PlanPart^b^	3.14	3.33	0.20***
ActRev^b^	3.92	3.90	− 0.02

Table 2 presents the descriptive statistics on the variables. Panel A reports on the full sample. Panel B reports on the change in the control problem from before to during the pandemic. Panel C compares the main variable means for low and high WFH (median split). *WFH* is the number of hours worked from home per week in the respective year indicated by the respondent. *ActCtrl* is the average of all action control constructs (i.e., *Standard, PlanPart,* and *ActRev*) in the respective year as indicated by the respondent. *Standard* is the average of all items related to standardization in the respective year as indicated by the respondent. *PlanPart* is the item related to planning participation in the respective year as indicated by the respondent. *ActRev* is the average of all items related to pre-action reviews in the respective year as indicated by the respondent. *TenureBoss* is the midpoint of the range indicated as tenure with the current supervisor in years by the respondent. *TenurePos* is the midpoint of the range indicated as tenure in the current position in years by the respondent. *TenureFirm* is the midpoint of the range indicated as tenure with the firm in years by the respondent. *Teamsize* is the total number of employees working in the same team/department. *TaskProg* is the average of all items related to task programmability in the respective year as indicated by the respondent. *Comm* is the score capturing the extent of informal communication along the hierarchy in the respective year as indicated by the respondent. *Social* is the average of all items related to socialization in the respective year indicated by the respondent. For detailed information on the survey questions see Appendix 1

^a^Indicates that we present the descriptive statistics of the variables before taking the natural logarithm as they are used in the regression models

^b^Indicates that we present the descriptive statistics of the variables based on the Likert scale before determining latent variables as they are used in the regression models

On average, employees work 16 h per week from home with a minimum of 0 h and a maximum of 50 h. The measures for action controls range between 1 and 7 with a mean of 3.9 for *ActCtrl*, 4.4 for *Standard*, 3.2 for *PlanPart*, and 3.9 for *ActRev*. Employees work for their current supervisor (in their current position) on average for 3.6 (5.6) years (*TenureBoss*, *TenurePos*) and they work for the firm for on average 6.9 years (*TenureFirm*). *Teamsize* ranges between 2 and 202 employees with a mean of 15.

Panel B shows the change in the control problem from before to during the pandemic. *WFH* has increased substantially from 5 h per week before the pandemic to 27 h per week during the pandemic (*p-*value < 0.01). We argue that—during the pandemic—employee actions are less observable; the information exchange between employees and their supervisors and colleagues is more difficult; the salience of social events that foster organizational identification and thus employees’ intrinsic motivation to work in the organization’s best interest is reduced. Consistently, we find a reduction in the extent to which supervisors can observe employees’ actions (*TaskProg*, *p*-value < 0.10). We also find a reduction in the extent to which informal communication is used to communicate along the hierarchy, but the difference is not statistically significant (*Comm*, *p*-value = 0.11). Finally, we find a statistically significant decrease in *Social* (*p*-value < 0.01), suggesting that the number of social events and programs has declined substantially from 2019 to 2021.

Panel C compares action controls for low and high WFH. We find that all action controls (i.e., *Standard*, *PlanPart*, *ActRev*) are stronger for high *WFH* than low *WFH*. The difference is statistically significant for *Standard* (*p*-value < 0.01), *PlanPart* (*p*-value < 0.01), and the aggregate measure of the action controls (*ActCtrl*, *p*-value < 0.01). However, we do not find a statistically significant difference for *ActRev*. While these comparisons are insightful, the bivariate nature does not account for further determinants of the use of MCs. We postpone a more detailed discussion of the association between WFH and action controls to the multiple regression analysis.^6^

### Main results

In Table 3, Panel A, we present the main findings of the OLS regression model related to Hypothesis H1. In Column (1), we operationalize action controls by the aggregate variable *ActCtrl*. In Columns (2) to (4), we operationalize action controls by the single constructs (i.e., *Standard*, *PlanPart*, and *ActRev*). We find a positive and statistically significant (*p*-value < 0.05) coefficient on *WFH* when using *ActCtrl* as the dependent variable. We also find a positive and statistically significant (*p*-value < 0.01) coefficient on *WFH* when using *Standard* and *PlanPart* as dependent variables. From 2019 to 2021, *WFH* has increased, on average, from 5 to 27 h. In terms of economic significance, an increase in *WFH* by 22 h is associated with an increase of 0.04 standard deviations in *ActCtrl*. The findings suggest that organizations strengthen action controls under WFH conditions, providing support for Hypothesis H1.

**Table 3:** OLS regressions on the association between WFH and the use of action controls (Hypothesis H1)

**Panel A: WFH and action controls**				
	(1)	(2)	(3)	(4)
Variables	ActCtrl	Standard	PlanPart	ActRev
WFH	0.03**	0.04***	0.04***	− 0.00
	(0.011)	(0.011)	(0.010)	(0.011)
TenureBoss	0.08**	0.08**	0.01	0.07*
	(0.038)	(0.037)	(0.039)	(0.039)
TenurePos	− 0.04	− 0.03	− 0.20***	0.02
	(0.046)	(0.046)	(0.049)	(0.046)
TenureFirm	− 0.08*	− 0.02	0.14***	− 0.17***
	(0.049)	(0.048)	(0.051)	(0.049)
Teamsize	− 0.01	− 0.00	− 0.01	− 0.02
	(0.039)	(0.039)	(0.040)	(0.039)
Division FE	YES	YES	YES	YES
Constant	0.04	0.06	− 0.08	0.04
	(0.126)	(0.123)	(0.120)	(0.121)
Observations	2,576	2,576	2,576	2,576
Adjusted R−squared	0.025	0.031	0.036	0.032
F-statistic	3.77***	4.97***	5.75***	4.03***

**Panel B: Pandemic and action controls**				
	(1)	(2)	(3)	(4)
Variables	ActCtrl	Standard	PlanPart	ActRev
Post	0.08***	0.11***	0.04***	0.02*
	(0.014)	(0.016)	(0.014)	(0.012)
TenureBoss	0.08**	0.07**	0.00	0.07*
	(0.038)	(0.038)	(0.039)	(0.039)
TenurePos	− 0.04	− 0.03	− 0.20***	0.02
	(0.046)	(0.046)	(0.049)	(0.046)
TenureFirm	− 0.08*	− 0.02	0.14***	− 0.17***
	(0.049)	(0.048)	(0.051)	(0.049)
Teamsize	− 0.01	− 0.00	− 0.01	− 0.02
	(0.039)	(0.039)	(0.040)	(0.039)
Division FE	YES	YES	YES	YES
Constant	0.05	0.07	− 0.02	0.02
	(0.124)	(0.122)	(0.119)	(0.120)
Observations	2,576	2,576	2,576	2,576
Adjusted R−squared	0.025	0.031	0.033	0.032
F−statistic	6.25***	8.03***	5.28***	4.28***

**Panel C: Pandemic, WFH, and action controls**				
	(1)	(2)	(3)	(4)
Variables	ActCtrl	Standard	PlanPart	ActRev
Post	0.06***	0.08***	0.04**	0.01
	(0.019)	(0.022)	(0.018)	(0.015)
WFHChange	− 0.07	− 0.05	0.12**	− 0.10*
	(0.056)	(0.056)	(0.056)	(0.056)
Post x WFHChange	0.05*	0.06*	0.01	0.01
	(0.028)	(0.033)	(0.028)	(0.025)
TenureBoss	0.08**	0.07**	0.00	0.07*
	(0.038)	(0.038)	(0.039)	(0.039)
TenurePos	− 0.04	− 0.03	− 0.20***	0.01
	(0.046)	(0.046)	(0.049)	(0.046)
TenureFirm	− 0.08*	− 0.02	0.14***	− 0.17***
	(0.049)	(0.048)	(0.051)	(0.049)
Teamsize	− 0.02	− 0.00	− 0.01	− 0.02
	(0.040)	(0.039)	(0.040)	(0.039)
Division FE	YES	YES	YES	YES
Constant	0.09	0.11	− 0.10	0.09
	(0.131)	(0.128)	(0.125)	(0.125)
Observations	2,576	2,576	2,576	2,576
Adjusted R-squared	0.025	0.03	0.036	0.034
F-statistic	5.42***	6.99***	4.95***	3.83***

Table 3 presents the OLS regression results on the association between WFH and the use of action controls (i.e., Hypothesis H1). Panel A presents the results on the association between WFH and action controls. Panel B presents the results on the association between the pandemic and action controls. Panel C presents the results on the association between the pandemic, WFH, and action controls. *ActCtrl* is the latent variable score obtained from construct validation of all action control constructs (i.e., *Standard, PlanPart*, and *ActRev*) in the respective year as indicated by the respondent. *Standard* is the latent variable score obtained from construct validation of all items related to standardization in the respective year as indicated by the respondent. *PlanPart* is the item related to planning participation in the respective year as indicated by the respondent. *ActRev* is the latent variable score obtained from construct validation of all items related to pre-action reviews in the respective year as indicated by the respondent. *WFH* is the natural logarithm of the number of hours worked from home per week in the respective year indicated by the respondent plus one. *TenureBoss* is the natural logarithm of the midpoint of the range indicated as tenure with the current supervisor in years by the respondent. *TenurePos* is the natural logarithm of the midpoint of the range indicated as tenure in the current position in years by the respondent. *TenureFirm* is the natural logarithm of the midpoint of the range indicated as tenure with the firm in years by the respondent. *Teamsize* is the natural logarithm of the total number of employees working in the same team/department. *Post* is an indicator variable equal to 1 if the year is 2021 and 0 for the year 2019. *WFHChange* is an indicator variable equal to 1 if the change in WFH is equal or higher than the median change in WFH, 0 otherwise. We include division fixed effects. The results are estimated by OLS and robust standard errors clustered at the employee level (reported in parentheses). *, **, *** indicate two-tailed significance at the 10 percent, 5 percent, and 1 percent levels, respectively. For detailed information on the survey questions see Appendix 1.

We do not find a statistically significant association between *WFH* and *ActRev*. This finding may be explained by the fact that action controls are not only beneficial because they address ensuing control problems, but their implementation is costly as they require the supervisor’s involvement. In particular, pre-action reviews are arguably more costly than standardizing processes. While the standardization of processes is associated with a one-time cost when the supervisor chooses the guidelines and procedures, pre-action reviews require more regular meetings and coordination between the supervisor and the employees.

Regarding the control variables, we find that employees’ tenure at the firm matters. For instance, we find a positive and statistically significant (*p*-value < 0.01) association between *TenureFirm* and *PlanPart* and we find a negative and statistically significant (*p*-value < 0.01) association between *TenureFirm* and *ActRev*. These findings suggest that supervisors more strongly rely on planning participation the longer the subordinate is working at the firm, while they rely less on pre-action reviews with subordinate tenure.

Since WFH significantly increased from 2019 to 2021 (see the descriptive statistics in Panel B of Table 2), the coefficient on *WFH* in Table 3 Panel A may (partly) capture the association between the pandemic and action controls (i.e., the change in action controls over time) rather than the association between WFH and action controls. For instance, supervisors may increase their use of action controls to account for the increase in uncertainty associated with the pandemic. To disentangle the underlying mechanism, we run two further analyses: (1) In Table 3 Panel B, we examine the association between the pandemic and action controls. In particular, we regress the measures of action controls on an indicator variable equal to 1 if the year is 2021, 0 for year 2019 (*Post*). (2) In Table 3 Panel C, we examine to what extent the pandemic and WFH is associated with action controls. More specifically, we regress the measures of action controls on *Post* and an indicator variable equal to 1 when the employee experienced a strong increase in WFH (i.e., an increase in WFH that is larger than the median increase of *WFH*), 0 otherwise (*WFHChange*). We add an interaction term between *Post* and *WFHChange* which captures whether there is a difference in action controls between employees who experienced a strong increase in WFH compared to those employees who experienced only a weak increase in WFH during compared to before the pandemic.

In Panel B, we find a positive and statistically significant coefficient on *Post* for all action control measures (*p*-value < 0.01 for *ActCtrl*, *Standard*, and *PlanPart*; *p*-value < 0.10 for *ActRev*), suggesting that action controls are stronger during compared to before the pandemic. In Panel C, we find a positive and statistically significant (*p*-value < 0.10) coefficient on the interaction between *Post* and *WFHChange* when using *ActCtrl* as the dependent variable. The results are consistent when using *Standard* as the dependent variable. For *PlanPart* and *ActRev*, we do not find a statistically significant coefficient on the interaction between *Post* and *WFHChange* (*p*-value > 0.10). The results in Panel C suggest that the increase in action controls during compared to before the pandemic is partly explained by those employees who experienced a significant increase in WFH. More generally, the findings suggest that action controls are stronger during the pandemic compared to before the pandemic and that the increase is partly explained by the shift toward WFH.

### Mechanism analysis: the role of control problems

In this subsection, to explore the mechanism behind the positive association between WFH and action controls, we examine the role of the control problem in explaining the association. In Table 4, we examine whether the severity of the control problem moderates the association between WFH and action controls. We differentiate between three dimensions of the control problem enhanced by the shift toward WFH: the lack of employee-task observability (*TaskProg*, Column (1)), communication limitations (*Comm*, Column (2)), and the decline in socialization (*Social*, Column (3)). In Column (4), we run the entire model. We add the same control variables as in Model (1). The results are estimated by OLS and robust standard errors clustered at the employee level.

**Table 4:** Mechanism analysis: OLS regressions on the role of control problems

	(1)	(2)	(3)	(4)
Variables	ActCtrl	ActCtrl	ActCtrl	ActCtrl
WFH	0.03***	0.02**	0.03***	0.03***
	(0.010)	(0.010)	(0.010)	(0.009)
TaskProg	0.43***			0.34***
	(0.025)			(0.025)
WFH x TaskProg	− 0.03**			− 0.03**
	(0.011)			(0.010)
Comm		0.20***		0.12***
		(0.017)		(0.015)
WFH x Comm		− 0.01*		− 0.01
		(0.008)		(0.008)
Social			0.35***	0.20***
			(0.027)	(0.026)
WFH x Social			− 0.01	0.00
			(0.012)	(0.012)
TenureBoss	0.04	0.08**	0.07**	0.04
	(0.034)	(0.036)	(0.036)	(0.032)
TenurePos	− 0.06	− 0.02	− 0.01	− 0.03
	(0.041)	(0.045)	(0.043)	(0.040)
TenureFirm	− 0.05	− 0.09*	− 0.08*	− 0.05
	(0.044)	(0.048)	(0.046)	(0.042)
Teamsize	− 0.01	− 0.01	− 0.03	− 0.02
	(0.036)	(0.038)	(0.037)	(0.034)
Division FE	YES	YES	YES	YES
Constant	0.06	0.09	0.07	0.05
	(0.112)	(0.118)	(0.117)	(0.107)
Observations	2,576	2,576	2,576	2,576
Adjusted R-squared	0.203	0.119	0.137	0.288
F-statistic	25.35***	15.08***	16.59***	33.55***

Table 4 presents the OLS regressions for the mechanism analysis on the role of control problems. *ActCtrl* is the latent variable score obtained from construct validation of all action control constructs (i.e., *Standard*, *PlanPart*, and *ActRev*) in the respective year as indicated by the respondent. *Standard* is the latent variable score obtained from construct validation of all items related to standardization in the respective year as indicated by the respondent. *PlanPart* is the item related to planning participation in the respective year as indicated by the respondent. *ActRev* is the latent variable score obtained from construct validation of all items related to pre-action reviews in the respective year as indicated by the respondent. *WFH* is the natural logarithm of the number of hours worked from home per week in the respective year indicated by the respondent plus one. *TaskProg* is the latent variable score obtained from construct validation of all items related to task programmability in the respective year as indicated by the respondent. *Comm* is the score capturing the extent of informal communication along the hierarchy in the respective year as indicated by the respondent. *Social* is the latent variable score obtained from construct validation of all items related to socialization in the respective year indicated by the respondent. *TenureBoss* is the natural logarithm of the midpoint of the range indicated as tenure with the current supervisor in years by the respondent. *TenurePos* is the natural logarithm of the midpoint of the range indicated as tenure in the current position in years by the respondent. *TenureFirm* is the natural logarithm of the midpoint of the range indicated as tenure with the firm in years by the respondent. *Teamsize* is the natural logarithm of the total number of employees working in the same team/department. We include division fixed effects. The results are estimated by OLS and robust standard errors clustered at the employee level (reported in parentheses)

*^,^**^,^*** indicate two-tailed significance at the 10%, 5%, and 1% levels, respectively. For detailed information on the survey questions see Appendix 1

Consistent with the main findings in Table 3, we find a positive and statistically significant association between *WFH* and *ActCtrl* in all columns (*p*-value < 0.01 in columns (1), (3), and (4) and *p-*value < 0.05 in Column (2)). More importantly, we find a negative and statistically significant (*p*-value < 0.05) coefficient on the interaction term of *WFH* and *TaskProg*. This finding suggests that the supervisor less strongly relies on action controls when employees more often work from home and employees’ task is more observable, i.e., when the control problem is less severe. Related, we find a negative and statistically significant (*p-*value < 0.10) coefficient on the interaction term of *WFH* and *Comm*, suggesting that the supervisor less strongly relies on action controls when employees more often work from home and there is a larger information exchange between supervisor and employee. We do not find that socialization (*Social*) explains the association between WFH and action controls.

When we combine all dimensions of the control problem in one model in Column (4), we find that the lack of observability of employees’ tasks is the dominating explanation of the positive association between WFH and action controls. We obtain qualitatively similar results when using standardization, planning participation, or pre-action reviews as dependent variables (untabulated analyses).

### Additional analysis

In the following, we explore the implications of the association between WFH and action controls and examine the association between WFH, action controls, and employee outcomes. We apply two measures of employee outcomes: (1) *MeetHr* captures the length of time employees spend in meetings per working day. It is measured as the average number of hours per day spent in meetings as indicated by the respondent.^7^ Employees spend on average 1.19 h per day in meetings, while there is some variance (std. dev. = 1.55). (2) *Focus* captures the extent to which employees can focus on their job measured on the scale 0–25%–50%–75%–100%. On average, employees indicated that they can focus on their job by 81%, while there is strong variance (std. dev. = 19).

To examine the association between WFH, action controls, and employee outcomes, we run similar regression models as in Table 3: (1) We regress *MeetHr* (*Focus*) on *WFH*, thereby examining the association between WFH and the extent to which employees spend time in meetings (can focus on their job) (Panel A of Table 5). (2) Since *WFH* captures both, variance in employee outcomes with WFH and with the pandemic, we then regress *MeetHr* (*Focus*) on *Post*, indicating how the extent to which employees spend time in meetings (can focus on their job) differs during compared to before the pandemic (Panel B of Table 5). *Post* is an indicator variable equal to 1 if the year is 2021 and 0 for the year 2019. (3) We explore to what extent the change in employee outcomes is associated with a change in WFH rather than the pandemic itself (e.g., an increase in uncertainty) by regressing *MeetHr* (*Focus*) on *Post* and *WFHChange* as well as the interaction between *Post* and *WFHChange* (Panel C of Table 5). *WFHChange* is an indicator variable equal to 1 if the change in WFH is equal to or higher than the median change in WFH, 0 otherwise. *WFHChange* splits the sample into employees who experienced a strong increase in WFH as compared to those employees who experienced a weak increase in WFH. Thus, while *Post* captures the difference in employee outcomes during as compared to before the pandemic, the interaction between *Post* and *WFHChange* explores whether the difference between employee ouctomes during as compared to before the pandemic is at least partly explained by the shift toward WFH.

To explore the moderating role of action controls, we replicate the analysis, splitting the sample into weak and strong action control (at the median of *ActCtrl*). We add the same control variables as in regression model (1). The results are estimated by OLS and robust standard errors clustered at the employee level. Table 5 reports on the findings of this analysis.

**Table 5:** Additional analysis: OLS regressions on the association between WFH, the use of action controls, and employee outcomes

**Panel A: WFH and employee outcomes**						
	(1a)	(1b)	(1c)	(2a)	(2b)	(2c)
Variables	MeetHr	MeetHr	MeetHr	Focus	Focus	Focus
Sample split	Full sample	Strong ActCtrl	Weak ActCtrl	Full sample	Strong ActCtrl	Weak ActCtrl
WFH	0.23***	0.20***	0.27***	2.21***	1.90***	2.55***
	(0.024)	(0.035)	(0.030)	(0.253)	(0.326)	(0.382)
TenureBoss	− 0.05	0.01	− 0.11*	0.98	1.07	0.29
	(0.043)	(0.060)	(0.059)	(0.648)	(0.811)	(0.975)
TenurePos	− 0.23***	− 0.14	− 0.30**	0.04	0.68	− 0.21
	(0.080)	(0.087)	(0.125)	(0.844)	(1.051)	(1.210)
TenureFirm	0.03	− 0.08	0.12	0.18	− 0.33	0.95
	(0.098)	(0.126)	(0.140)	(0.877)	(1.080)	(1.310)
Teamsize	0.13	0.29	− 0.02	1.12*	1.57*	0.72
	(0.103)	(0.178)	(0.082)	(0.644)	(0.834)	(0.957)
Division FE	YES	YES	YES	YES	YES	YES
Constant	0.61***	0.32	0.90***	70.48***	71.37***	69.25***
	(0.201)	(0.308)	(0.233)	(2.032)	(2.532)	(3.124)
Observations	2,390	1,195	1,195	2,390	1,195	1,195
Adjusted R-squared	0.140	0.141	0.158	0.0405	0.0412	0.0387
F-statistic	35.56***	19.22***	19.65***	9.20***	5.25***	5.17**

**Panel B: Pandemic and employee outcomes**						
	(1a)	(1b)	(1c)	(2a)	(2b)	(2c)
Variables	MeetHr	MeetHr	MeetHr	Focus	Focus	Focus
Sample split	Full sample	Strong ActCtrl	Weak ActCtrl	Full sample	Strong ActCtrl	Weak ActCtrl
Post	0.59***	0.58***	0.62***	6.04***	4.78***	7.00***
	(0.037)	(0.055)	(0.056)	(0.644)	(0.877)	(0.978)
TenureBoss	− 0.06	0.00	− 0.11*	0.91	1.00	0.24
	(0.045)	(0.061)	(0.061)	(0.643)	(0.815)	(0.961)
TenurePos	− 0.24***	− 0.15*	− 0.32**	− 0.13	0.52	− 0.41
	(0.083)	(0.089)	(0.130)	(0.834)	(1.060)	(1.189)
TenureFirm	0.04	-0.07	0.13	0.31	− 0.19	1.08
	(0.101)	(0.127)	(0.145)	(0.871)	(1.089)	(1.299)
Teamsize	0.14	0.28	0.01	1.16*	1.48*	0.95
	(0.102)	(0.177)	(0.084)	(0.635)	(0.825)	(0.950)
Division FE	YES	YES	YES	YES	YES	YES
Constant	0.78***	0.47	1.08***	71.91***	73.20***	70.41***
	(0.228)	(0.353)	(0.262)	(1.973)	(2.485)	(3.063)
Observations	2,390	1,195	1,195	2,390	1,195	1,195
Adjusted R-squared	0.125	0.137	0.127	0.0346	0.0328	0.0320
F-statistic	36.68***	19.55***	20.23***	9.92***	4.68***	5.51***

**Panel C: Pandemic, WFH, and employee outcomes**						
Variables	(1a)	(1b)	(1c)	(2a)	(2b)	(2c)
	MeetHr	MeetHr	MeetHr	Focus	Focus	Focus
Sample split	Full sample	Strong ActCtrl	Weak ActCtrl	Full sample	Strong ActCtrl	Weak ActCtrl
Post	0.52***	0.56***	0.50***	0.21	0.05	0.06
	(0.049)	(0.067)	(0.078)	(0.822)	(1.116)	(1.296)
WFHChange	0.16**	0.24**	0.10	− 4.92***	− 3.47**	− 5.92***
	(0.075)	(0.104)	(0.104)	(1.147)	(1.575)	(1.641)
Post x WFHChange	0.15**	0.04	0.22**	11.63***	9.90***	13.17***
	(0.074)	(0.112)	(0.108)	(1.245)	(1.719)	(1.872)
TenureBoss	− 0.06	− 0.01	− 0.11*	0.89	0.93	0.24
	(0.044)	(0.062)	(0.060)	(0.642)	(0.814)	(0.962)
TenurePos	− 0.23***	− 0.14	− 0.31**	− 0.10	0.62	− 0.41
	(0.084)	(0.088)	(0.133)	(0.838)	(1.065)	(1.204)
TenureFirm	0.04	− 0.07	0.13	0.32	− 0.18	1.08
	(0.100)	(0.126)	(0.146)	(0.874)	(1.091)	(1.308)
Teamsize	0.15	0.30*	0.02	1.21*	1.56*	1.01
	(0.102)	(0.175)	(0.086)	(0.637)	(0.832)	(0.951)
Division FE	YES	YES	YES	YES	YES	YES
Constant	0.66***	0.31	1.00***	74.21***	74.50***	73.45***
	(0.231)	(0.341)	(0.292)	(2.065)	(2.708)	(3.109)
Observations	2,390	1,195	1,195	2,390	1,195	1,195
Adjusted R-squared	0.130	0.142	0.132	0.0573	0.0527	0.0571
F-statistic	35.23***	18.55***	19.26***	13.69***	6.32***	7.86***

Table 5 reports the regression results of an additional analysis on the association between WFH, action controls, and employee outcomes. Panel A presents the results on the association between WFH and employee outcomes. Panel B presents the results on the association between the pandemic and employee outcomes. Panel C presents the results on the association between the pandemic, WFH, and employee outcomes. In columns (1a) and (2a), we present the results on the full sample. In columns (1b) to (1c) and (2b) to (2c), we split the sample at the median of *ActCtrl. MeetHr* is the average number of hours per day spent in meetings as indicated by the respondent. *Focus* is the indicated extent to which the respondent can focus on the job as indicated by the respondent. *WFH* is the natural logarithm of the number of hours worked from home per week in the respective year indicated by the respondent plus one. *Post* is an indicator variable equal to 1 if the year is 2021 and 0 for the year 2019. *WFHChange* is an indicator variable equal to 1 if the change in WFH is equal or higher than the median change in WFH, 0 otherwise. *TenureBoss* is the natural logarithm of the midpoint of the range indicated as tenure with the current supervisor in years by the respondent. *TenurePos* is the natural logarithm of the midpoint of the range indicated as tenure in the current position in years by the respondent. *TenureFirm* is the natural logarithm of the midpoint of the range indicated as tenure with the firm in years by the respondent. *Teamsize* is the natural logarithm of the total number of employees working in the same team/department. We include division fixed effects. The results are estimated by OLS and robust standard errors clustered at the employee level (reported in parentheses). *, **, *** indicate two-tailed significance at the 10 percent, 5 percent, and 1 percent levels, respectively. For detailed information on the survey questions see Appendix 1

In Panel A, we find a positive and statistically significant (*p*-value < 0.01) coefficient on *WFH* when using *MeetHr* and *Focus* as dependent variables, respectively. These findings suggest that employees who more often work from home spend more time in meetings and can better focus on their job. Although the coefficient on *WFH* is slightly higher for the sample of weak action controls (Column (1c)) compared to the sample of strong action controls (Column (1b)) when using *MeetHr* as the dependent variable, there is no statistically significant difference between the coefficients (*p*-value = 0.11). Likewise, although the coefficient on *WFH*, when using *Focus* as the dependent variable, is slightly higher for the sample of weak action controls (Column (1c)) compared to the sample of strong action controls (Column (1b)), there is no statistically significant difference between the coefficients (*p*-value = 0.19).

In Panel B, we find a positive and statistically significant (*p*-value < 0.01) coefficient on *Post* when using *MeetHr* and *Focus* as dependent variables, respectively. This finding suggests that employees spend more time in meetings and can better focus on their job during compared to before the pandemic. Since the average number of overtime hours per week did not statistically significantly (*p*-value > 0.10) change during compared to before the pandemic (results untabulated), the remaining working hours per day likely decreased. When using *MeetHr* as the dependent variable, there is no statistically significant difference between the coefficients on *WFH* for the sample of weak action controls (Column (1c)) compared to the sample of strong action controls (Column (1b)) (*p*-value > 0.10). Likewise, although the coefficient on *Post* when using *Focus* as dependent variable is higher for the sample of weak action controls (Column (1c)) compared to the sample of strong action controls (Column (1b)), there is no statistically significant difference between the coefficients (*p*-value = 0.15).

In Panel C, we find a positive and statistically significant (*p*-value < 0.01) coefficient on *Post* when using *MeetHr* as dependent variable. More importantly, we find a positive and statistically significant (*p-*value < 0.05) coefficient on the interaction between *Post* and *WFHChange* in the full sample (column (1a)). This finding suggests that the increase in the average number of hours employees spend in meetings during compared to before the pandemic is partly explained by an increase in WFH. However, this is only true for employees exposed to weak action controls. That is, we find a positive and statistically significant (*p*-value < 0.05) coefficient on the interaction between *Post* and *WFHChange* for the sample with weak action controls (Column (1c)), whereas the coefficient is statistically insignificant for strong action controls (Column (1b)). This finding suggests that WFH increases the average number of hours employees spend in meetings per day for those employees exposed to weak action controls, but not for those employees exposed to strong action controls.

When using *Focus* as the dependent variable (columns (2a)–(2c)), we do not find a statistically significant (*p*-value > 0.10) coefficient on *Post*, suggesting that there is no difference in the extent to which employees can focus on their job during compared to before the pandemic. However, we find a positive and statistically significant (*p*-value < 0.01) coefficient on the interaction between *Post* and *WFHChange*. This finding suggests that employees are better able to focus on their job during compared to before the pandemic when they experience a stronger increase in WFH. We find that the coefficient on the interaction between *Post* and *WFHChange* is higher for the sample of weak action controls as compared to the sample of strong action controls, but the difference in coefficients is not statistically significant (*p-*value > 0.10).

Overall, the findings suggest that WFH induced by the pandemic increased the time employees spend in meetings, likely decreasing the remaining working hours per day, and increased the extent to which employees can focus on their job. Furthermore, we provide weak evidence for a moderating role of action controls. In particular, the findings suggest that, while strong action controls mitigate the number of hours working-from-home employees spend in meetings, they may also hinder the extent to which employees can focus on their job when working from home. The latter finding suggests that, by specifying boundaries to acceptable behavior or by implementing formal approval procedures (Simons 1995), strong action controls may limit the discretion of employees, interrupt employees’ task fulfillment, and ultimately reduce employees’ focus.

In terms of economic significance, in Panel C, average meeting hours per day increased by 31 min in 2021 as compared to 2019 for those employees who experienced a small or no increase in WFH (smaller than or equal to the median change in WFH). For those employees who experienced a strong increase in WFH (larger than the median change in WFH), average meeting hours per day increased by 50 min in 2021 as compared to 2019. Finally, we show that job focus for employees who experienced a small or no increase in WFH (smaller or equal to the median change in WFH) increases by 0.2% points. In comparison, it increases by 7% points for those employees who experienced a strong increase in WFH (larger than the median change in WFH).

## Conclusion

This study examines how the Covid-19 pandemic and the corresponding shift toward WFH affects the design of MCs. The findings suggest that WFH amplifies control problems within organizations. In particular, employees indicate that their tasks are less observable, arguably decreasing their supervisor’s monitoring capabilities. Moreover, socialization processes that align employees with the organization’s objectives are less prevalent, arguably reducing organizational identification and employees’ motivation. We provide evidence that organizations adjust the action controls when employees more often work from home. Specifically, there is a higher standardization of processes and a stronger involvement in strategic planning processes for employees who work from home. These results are partly explained by the supervisor’s lack of observability of employees’ tasks.

We also examine the association between WFH and employee outcomes and the potentially moderating role of MC practices. We document that the number of hours employees spend in meetings is significantly higher in 2021 as compared to 2019, which is partly explained by the strong increase in WFH from 2019 to 2021. Interestingly, this association is only statistically significant in the subsample of weak action controls. Thus, strong action controls seem to reduce the necessity for time-consuming meetings. In addition, we find that employees can better focus on their job in 2021 as compared to 2019 when they experienced a strong increase in WFH. Overall, the study adds to the literature by exploring the association between WFH, MCs, and employee outcomes.

The study is subject to limitations. First, the design of this study does not allow to draw causal inferences on the effect of WFH on the use of action controls. However, we provide first evidence that there is a positive association between WFH and action controls. Second, we focus on action controls rather than exploring the full set of MC practices that is available to supervisors, including result controls, personnel controls, cultural controls, and the combination thereof. We focus the analysis on action controls because of the organization’s ability to adjust action controls in the short-term, whereas adjusting result, personnel, and cultural controls, respectively, is arguably more time-consuming. Future research may investigate mid- or long-term adjustments in a firm’s MC elements (including result, personnel, and cultural controls). We also investigate action control practices as a package rather than a system of interrelated practices. According to the systems approach of contingency theory (Drazin and Van de Ven 1985), organizations assure internally consistent MCs, implying interdependent MC practices (Grabner and Moers 2013). Future research may investigate potential complementary or substitute relations between the separate elements of action control. Third, we ask respondents to answer questions related to the years 2019 and 2021 at one point in time, potentially raising a memory bias. Future research may examine the long-term consequences of WFH on the use of MC practices.

## Data Availability

Data is from a proprietary source and subject to confidentiality agreements.

## Appendix 1

**Table:**

Variable name	Construct	Survey questions	Time period	Response scale	Variable measurement
WFH	Working from home	How often did you work from home in 2019?	*as indicated in the question*	Average number of hours per week	Natural logarithm of the number of hours worked from home per week in the respective year as indicated by the respondent plus one
		How often did you work from home from March to May 2020?			
		How often do you currently [2021] work from home?			
Standard	Standardization (Bedford and Malmi 2015)	To what extent are your work activities determined by standardized procedures or processes?	2019/2021	1 for "very small extent" and 7 for "very high extent"	Latent variable score obtained from construct validation of all items related to standardization in the respective year as indicated by the respondent
		To what extent are the activities between organizational units (e.g., teams, departments) coordinated by	–		
		– pre-planning of activities.	2019/2021		
		– formal or informal standards, programs or procedures.			
PlanPart	Planning participation (Bedford and Malmi 2015)	To what extent are you involved in the strategic planning processes of your organizational unit (e.g., team, department)?	2019/2021	1 for "very small extent" and 7 for "very high extent"	Item related to planning participation in the respective year as indicated by the respondent
ActRev	Pre-action reviews (Bedford and Malmi 2015)	To what extent do you coordinate your procedure at projects/tasks with your supervisor in advance?	2019/2021	1 for "very small extent" and 7 for "very high extent"	Latent variable score obtained from construct validation of all items related to pre-action reviews in the respective year as indicated by the respondent
		How detailed are the reports or plans your supervisor requires from you before you undertake specific projects/tasks?		1 for "not very detailed" and 7 for "very detailed"	
ActCtrl	Action control				Latent variable score obtained from construct validation of all action control constructs (i.e., Standard, PlanPart, and ActRev)
TenureBoss	Tenure with the supervisor	How long have you been working under your current supervisor? (in years)	2021	Less than 1 year, 1–3 years, 4–6 years, 6–10 years, more than 10 years	Natural logarithm of the midpoint of the range indicated as tenure with the current supervisor in years by the respondent
TenurePos	Tenure in the current position	How long have you been working in your current position? (in years)	2021	Less than 1 year, 1–3 years, 4–6 years, 6–10 years, more than 10 years	Natural logarithm of the midpoint of the range indicated as tenure in the current position in years by the respondent
TenureFirm	Tenure in the firm	How long have you been working for the company? (in years)	2021	Less than 1 year, 1–3 years, 4–6 years, 6–10 years, more than 10 years	Natural logarithm of the midpoint of the range indicated as tenure with the firm in years by the respondent
Teamsize	Size of the team	*Determination based on job position identifier*			Natural logarithm of the total number of employees working in the same team/department
TaskProg	Task program-mability (Bedford and Malmi 2015)	Please indicate to what extent you agree or disagree with the following statements:	–	–	Latent variable score obtained from construct validation of all items related to task programmability in the respective year as indicated by the respondent
		– The actions you take to achieve results are visible to your supervisor.	2019/2021	1 for "strongly disagree" and 7 for "strongly agree"	
		– Your supervisor can distinguish effective and ineffective employees by observing their actions.			
		– The relationship between the actions you take and the subsequent outcomes are well known to your supervisor.			
Comm	Informal communication (Kober et al., 2007)	To what extent is informal communication used in passing information up and down the hierarchy?	2019/2021	1 for "very small extent" and 7 for "very high extent"	Score capturing the extent of informal communication along the hierarchy in the respective year as indicated by the respondent
Social	Socialization (Bedford and Malmi 2015)	To what extent are…	–	–	Latent variable score obtained from construct validation of all items related to socialization in the respective year indicated by the respondent
		– training and development processes used to reinforce the objectives, expectations and norms of the company?	2019/2021	1 for "very small extent" and 7 for "very high extent"	
		– social events and functions used to develop and maintain commitment to the company?			
		– mentoring, orientation and induction programs used to acclimatize new employees to acceptable behaviors, routines and norms?			
MeetHr	Time employees spend in meetings per working day	How many meetings do you have on average per day?	2019/2021	Average number of meetings per day	Average number of hours per day spent in meetings in the respective year as indicated by the respondent
		How long does a meeting take on average in minutes?		Average duration per meeting	
Focus	Employee's focus on the job	To what extent can you concentrate on your work?	2019/2021	0%, 25%, 50%, 75%, 100%	Extent to which the respondent can focus on the job in the respective year as indicated by the respondent

Appendix 1 provides an overview on the main variables. It reports information on the respective survey questions, response scales, and variable measurement.

## Appendix 2

To evaluate the validity of the latent constructs, we follow Kennedy and Widener (2019) and Bedford et al. (2022), among others, and use the measurement model of a Partial Least Squares Structural Equation Modeling (PLS-SEM) estimation. More specifically, to assess construct validity, we follow Sarstedt et al. (2019) and apply a disjoint two-stage PLS-SEM approach. Tables A.1 and A.2 present the results of the measurement model of the first and second stage PLS-SEM, respectively. We obtain a factor score from the measurement model of the PLS-SEM for each construct.

To assess construct validity for reflective constructs, we examine unidimensionality, construct reliability, and convergent and discriminant validity (Bedford and Speklé 2018; Mehmetoglu and Venturini 2021). Regarding unidimensionality, the eigenvalues obtained from a (untabulated) principal component analysis indicate that the respective items can be explained by a single construct. Dillon–Golstein’s rho (DG rho) exceeds the threshold of 0.70, indicating construct reliability (Hair et al. 2018). To assess convergent validity, we consider factor loadings and average variance extracted (AVE). All items load strongly on their respective construct and the standardized loadings are statistically significant. The AVE lies above the recommended level of 0.5 (Hair et al. 2018). Regarding discriminant validity, the AVE is larger than the squared correlation with any other construct in the measurement model (Fornell and Larcker 1981).

For formative constructs, we examine content validity, the statistical significance of the item weights, and multicollinearity between construct items (Mehmetoglu and Venturini 2021). As we rely on pre-established formative constructs from the literature, we claim a sufficient degree of content validity (see Bedford and Malmi 2015 for a detailed discussion)*.* All item weights are statistically significant at the 1% level. Regarding multicollinearity, all variance inflation factors (untabulated) are below the recommended threshold of 3.33 (Diamantopoulos and Siguaw 2006; Petter et al. 2007).

**Table A.1:** Measurement Model of the first stage PLS-SEM

Panel A: Construct Reliability and Convergent Validity of lower-order constructs			
	(1)	(2)	(3)
	Standard	PlanPart	ActRev
	Reflective	Single-item	Formative
Standard1	0.58***		
Standard2	0.92***		
Standard3	0.89***		
PlanPart1		1.0***	
ActRev1			0.80***
ActRev2			0.95***
DG rho	0.85		

Panel B: Discriminant Validity of lower-order constructs			
	(1)	(2)	(3)
	Standard	PlanPart	ActRev
Standard	1.00		
PlanPart	0.04	1.00	
ActRev	0.10	0.06	1.00
AVE	0.66		

Table A.1 reports the measurement model of the first stage PLS-SEM to evaluate the construct reliability and construct validity of the lower-order constructs. Panel A reports the standardized factor loadings for the latent constructs *Standard, PlanPart, ActRev* as well as Dillon-Goldstein's rho (DG rho) as a measure for construct reliability for the reflective construct *Standard*. Panel B reports the correlations among the latent constructs in order to assess how uniquely the construct's indicators represent the construct, i.e., comparison of shared variance within a construct and the variance between the constructs. Additionally, the average variance extracted (AVE) is displayed. The standard errors were estimated with a bootstrap procedure with 10,000 replications. For detailed information on the survey questions see Appendix 1

**Table A.2:** Measurement Model of the second stage PLS-SEM

Panel A: Construct Reliability and Convergent Validity of higher-order and additional constructs			
	(1)	(2)	(3)
	ActCtrl	TaskProg	Social
	formative	reflective	formative
Standard	0.81***		
ActRev	0.80***		
PlanPart	0.43***		
TaskProg1		0.85***	
TaskProg2		0.86***	
TaskProg3		0.83***	
Social1			0.88***
Social2			0.31***
Social3			0.86***
DG rho		0.88	

Panel B: Discriminant Validity of higher-order construct and additional constructs			
	(1)	(2)	(3)
	ActCtrl	TaskProg	Social
ActCtrl	1.00		
TaskProg	0.18	1.00	
Social	0.12	0.06	1.00
AVE		0.72	

Table A.2 reports the measurement model of the second stage PLS-SEM to evaluate the construct reliability and construct validity of the higher-order and additional latent constructs. Panel A reports the standardized factor loadings for the higher-order construct *ActCtrl* and the additional construct *TaskProg* and *Social* as well as Dillon-Goldstein's rho (DG rho) as a measure for construct reliability. Panel B reports the correlations among the latent constructs in order to assess how uniquely the construct's indicators represent the construct, i.e., comparison of shared variance within a construct and the variance between the constructs. Additionally, the average variance extracted (AVE) is displayed. The standard errors were estimated with a bootstrap procedure with 10,000 replications. For detailed information on the survey questions see Appendix 1

## Appendix 3

**Table:**

Pearson Correlation Coefficients												
Variables	(1)	(2)	(3)	(4)	(5)	(6)	(7)	(8)	(9)	(10)	(11)	(12)
(1) WFH	1.00											
(2) ActCtrl	0.03*	1.00										
(2) Standard	0.05**	0.81***	1.00									
(3) PlanPart	0.06***	0.43***	0.22***	1.00								
(4) ActRev	− 0.01	0.80***	0.33***	0.24***	1.00							
(6) TenureBoss	− 0.06***	0.02	0.03	− 0.04*	0.02	1.00						
(7) TenurePos	− 0.04**	− 0.04**	− 0.02	− 0.09***	− 0.03	0.51***	1.00					
(8) TenureFirm	− 0.02	− 0.06***	− 0.03	− 0.01	− 0.07***	0.46***	0.74***	1.00				
(9) Teamsize	0.03	− 0.03*	− 0.02	− 0.00	− 0.04**	0.00	− 0.06***	0.00	1.00			
(10) TaskProg	− 0.01	0.43***	0.33***	0.16***	0.37***	0.06***	0.02	− 0.01	− 0.02	1.00		
(11) Comm	0.02	0.32***	0.26***	0.21***	0.24***	0.00	− 0.03	− 0.02	− 0.02	0.19***	1.00	
(12) Social	− 0.03	0.34***	0.30***	0.19***	0.24***	0.00	− 0.06***	− 0.06***	-0.00	0.25***	0.33***	1.00

Appendix 3 presents the Pearson correlation between the main variables. *WFH* is the natural logarithm of the number of hours worked from home per week in the respective year indicated by the respondent plus one. *ActCtrl* is the latent variable score obtained from construct validation of all action control constructs (i.e., *Standard, PlanPart,* and *ActRev)* in the respective year as indicated by the respondent. *Standard* is the latent variable score obtained from construct validation of all items related to standardization in the respective year as indicated by the respondent. *PlanPart* is the item related to planning participation in the respective year as indicated by the respondent. *ActRev* is the latent variable score obtained from construct validation of all items related to pre-action reviews in the respective year as indicated by the respondent. *TenureBoss* is the natural logarithm of the midpoint of the range indicated as tenure with the current supervisor in years by the respondent. *TenurePos* is the natural logarithm of the midpoint of the range indicated as tenure in the current position in years by the respondent. *TenureFirm* is the natural logarithm of the midpoint of the range indicated as tenure with the firm in years by the respondent. *Teamsize* is the natural logarithm of the total number of employees working in the same team/department. *TaskProg* is the latent variable score obtained from construct validation of all items related to task programmability in the respective year as indicated by the respondent. *Comm* is the score capturing the extent of informal communication along the hierarchy in the respective year as indicated by the respondent. *Social* is the latent variable score obtained from construct validation of all items related to socialization in the respective year indicated by the respondent. For detailed information on the survey questions see Appendix 1

## Footnotes

1 We acknowledge the possibility for a positive association between WFH and the use of result controls. Following Groen et al. (2018), supervisors might substitute the lack of observability of employees’ actions by more strongly relying on result controls.

2 Before the pandemic, WFH was restricted in the sample firm due to a lack of technical infrastructure and contractual agreements with the workers’ council. As such, WFH before the pandemic was almost negligible (roughly 5 h per week). During the pandemic (January 19, 2021 and June 30, 2021), WFH was obligatory in Germany—where possible—due to governmental regulation (Corona Datenplattform 2021). However, we still observe a substantial variation in WFH even for the year 2021 (standard deviation 13.30).

3 We collect all responses at a single point in time (March and April 2021), which means we asked in 2021 for the current period and retrospectively for 2019. Therefore, the results could be subject to a memory bias such as recency or consistency bias. For example, the consistency bias captures the instinctual desire to remain consistent with prior beliefs (Cacioppo et al. 2002). Jaspers et al. (2009) and Hipp et al. (2020) show that the change between past and present is likely underestimated when using retrospective survey questions. Therefore, a memory bias decreases variation in the results, leading to a reduction in effect sizes.

4 We replicate the OLS regression model (1) for alternative variable specifications in two (untabulated) robustness checks. First, we rely on the measures *ActCtrl*, *Standard*, *PlanPart*, and *ActRev* obtained when taking the average of all items related to the single constructs (i.e., when using equal weighting). Second, we use each item of the constructs *Standard*, *PlanPart*, and *ActRev* in a separate OLS regression rather than aggregating the items to a construct score. The results of the robustness checks are largely consistent.

5 Since the variables are based on employees’ survey responses, we rely on the employees’ perception of control practices. To address potential concerns associated with perception measures, we use survey responses from 292 supervisors on the actual use of action controls to examine the association between the supervisors’ *use* of action controls (based on the supervisors’ responses to the survey questions) and employees’ *perception* of action controls. The (untabulated) findings show a statistically significant (*p*-value < 0.01) association between supervisors’ and employees’ survey responses, respectively. This suggests that employees’ perception of action controls is a good proxy for supervisors’ use of action controls.

6 For Pearson correlation coefficients, please see Appendix 3.

7 When constructing the variable *MeetHr*, we remove all observations where the number of hours spent in meetings per day exceeds 24 h (i.e., *MeetHr* > 24). The results of the additional analysis are consistent when we alternatively winsorize the variables at the 99th percentile or use the raw data.

## References

[CR1] Abernethy MA, Bouwens J, Van Lent L (2004) Determinants of control system design in divisionalized firms. Account Rev 79(3):545–570

[CR2] Alipour J-V, Fadinger H, Schmyk J (2020) My home is my castle. ifo Working Papers

[CR3] Allen TD, Golden TD, Shockley KM (2015) How effective is telecommuting? Assessing the status of our scientific findings. Psychol Sci Publ Interest 16(2):40–68 10.1177/1529100615593273 26403188

[CR4] Angelici M, Profeta P (2020) Smart-working:work flexibility without constraints. CESifo Working Paper, No. 8165, Center for Economic Studies and Ifo Institute

[CR5] Anthony R, Govindarajan V (2004) Management control systems, 11th edn. McGraw-Hill, New York

[CR6] Armstrong JS, Overton TS (1977) Estimating nonresponse bias in mail surveys. J Mark Res 14(3):396–402

[CR7] Arya A, Fellingham J, Glover J (1997) Teams, repeated tasks, and implicit incentives. J Account Econ 23:7–23

[CR8] Ashforth BE, Kreiner GE, Fugate M (2000) All in a day’s work: boundaries and micro role transition. Acad Manag Rev 25(3):472–491

[CR9] Barrero JM, Bloom N, Davis S (2021) Why working from home will stick. Working Paper. https://www.nber.org/papers/w28731

[CR10] Bedford DS, Malmi T (2015) Configurations of control: an exploratory analysis. Manag Account Res 27:2–26

[CR11] Bedford DS, Malmi T, Sandelin M (2016) Management control effectiveness and strategy: an empirical analysis of packages and systems. Acc Organ Soc 51:12–28

[CR12] Bedford DS, Speklé RF (2018) Construct validity in survey-based management accounting and control research. J Manag Account Res 30(2):23–58

[CR13] Bedford DS, Speklé RF, Widener SK (2022) Budgeting and employee stress in times of crisis: evidence from the Covid-19 pandemic. Acc Organ Soc: 1013466

[CR14] Bisbe J, Otley DT (2004) The effects of the interactive use of management control systems on product innovation. Acc Organ Soc 29(8):709–737

[CR15] Bischof J, Dörrenberg P, Rostam-Afschar D, Simons D, Voget J (2022) The German Business Panel : evidence on accounting and business taxation. Working Paper

[CR16] Bischof J, Simons D, Voget J, Dörrenberg P, Rostam-Afschar D, Buhlmann F, Akari P, Arnemann L, Eble F, Gharbi S, Karlsson C (2020) German business Panel: Wirkung und Zielgenauigkeit der staatlichen Hilfen für Firmen in der Krise. August 2020: 1–15

[CR17] Bloom N, Liang J, Roberts J, Ying ZJ (2015) Does working from home work? Evidence from a Chinese experiment. Q J Econ 130(1):165–218

[CR18] Bloom N, Fletcher RS, Bloom N (2021) The impact of covid-19 on US firms. National Bureau of Economic Research Working Paper Series

[CR19] Bonet R, Salvador F (2017) When the boss is away: manager-worker separation and worker performance in a multisite software maintenance organization. Organ Sci 28(2):244–261

[CR20] Brownson K (2004) The benefits of a work-at-home program. Health Care Manager 23(2):141–144 15192994 10.1097/00126450-200404000-00007

[CR21] Bundesbank (2021). Einschätzungen und Erwartungen von Unternehmen in der Pandemie: Erkenntnisse aus dem Bundesbank-Online-Panel-Firmen. Monatsbericht 2021: 35–60

[CR22] Cacioppo JT, Berntson GG, Adolphs R, Carter CS, Davidson RJ, McClintock MK, McEwen BS, Meaney MJ, Schacter DL, Sternberg EM, Suomi SS, Taylor SE (eds) (2002) Foundations in social neuroscience. MIT Press

[CR23] Chenhall RH (2003) Management control systems design within its organizational context: findings from contingency-based research and directions for the future. Acc Organ Soc 28:127–168

[CR24] Chin WW (2010) How to write up and report PLS analyses. In: Esposito Vinzi V, Chin WW, Henseler J, Wang H (eds) Handbook of partial least squares: concepts, methods and applications. Springer, pp 655–690

[CR25] Coch L, French JR Jr (1948) Overcoming Resistance to Change. Hum Relat 1(4):512–532

[CR26] Corona Datenplattform (2021). Themenreport 02, Homeoffice im Verlauf der Corona-Pandemie, Ausgabe Juli 2021, Bonn

[CR27] Daft RL, Macintosh NB (1984) The nature and use of formal control systems for management control and strategy implementation. J Manag 10(1):43–66

[CR28] DeFilippis E, Impink SM, Singell M, Polzer JT, Sadun R (2020) Collaborating during coronavirus: the impact of covid-19 on the nature of work. National Bureau of Economic Research Working Paper Series

[CR29] De Menezes LM, Kelliher C (2011) Flexible working and performance: a systematic review of the evidence for a business case. Int J Manag Rev 13(4):452–474

[CR31] Diamantopoulos A, Siguaw JA (2006) Formative versus reflective indicators in organizational measure development: a comparison and empirical illustration. Br J Manag 17(4):263–282

[CR32] DIW Berlin (2016) Home Office: Möglichkeiten werden bei weitem nicht ausgeschöpft, Karl Brenke. DIW Wochenbericht Nr. 5.2016

[CR33] Dulebohn JH, Hoch JE (2017) Virtual teams in organizations. Hum Resour Manag Rev 27(4):569–574

[CR34] Eversole BAW, Venneberg DL, Crowder CL (2012) Creating a flexible organizational culture to attract and retain talented workers across generations. Adv Dev Hum Resour 14(4):607–625

[CR35] Felstead A, Jewson N, Walters S (2003) Managerial control of employees working at home. Br J Ind Relat 41(2):241–264

[CR36] Forbes (2021) Working from home In 2021: The Legal Problems.

[CR37] Fornell C, Larcker DF (1981) Evaluating structural equation models with unobservable variables and measurement error. J Mark Res 18(1):39–50

[CR38] Gajendran RS, Harrison DA (2007) The good, the bad, and the unknown about telecommuting: meta-analysis of psychological mediators and individual consequences. J Appl Psychol 92(6):1524-1541 18020794 10.1037/0021-9010.92.6.1524

[CR39] Gerdin J (2005) The impact of departmental interdependencies and management accounting system use on subunit performance: a second look. Eur Account Rev 14(2):335–340

[CR40] Gerdin J, Johansson T, Wennblom G (2019) The contingent nature of complementarity between results and value-based controls for managing company-level profitability: a situational strength perspective. Acc Organ Soc 79:101058

[CR41] Gibbs M, Mengel F, Siemroth C (2022) Work from Home and productivity: evidence from personnel and analytics data on IT professionals. J Polit Econ Microecon (forthcoming)

[CR42] Golden TD, Veiga JF, Dino RN (2008) The impact of professional isolation on teleworker job performance andturnover intentions: does time spent teleworking, interacting face-to-face, or having access to communication-enhancing technology matter? J Appl Psychol 93:1412–1421 19025257 10.1037/a0012722

[CR150] Grabner I, Moers F (2013) Management control as a system or a package? Conceptual and empirical issues. Account Organ Soc 38(6–7):407–419

[CR43] Grabner I, Posch A, Wabnegg M (2018) Materializing innovation capability: a management control perspective. J Manag Account Res 30(2):163–185

[CR44] Greer TW, Payne SC (2014) Overcoming telework challenges: outcomes of successful telework strategies. Psychol Manager J 17(2):87–111

[CR45] Harrison GL, McKinnon JL (1999) Cross-cultural research in management control systems design: a review of the current state. Acc Organ Soc 24(5–6):483–506

[CR46] Hair J, Anderson R, Tatham R, Black W (2018) Multivariate Data Analysis, 8th edn. Prentice Hall, New Jersey

[CR47] Heinicke A, Guenther TW, Widener SK (2016) An examination of the relationship between the extent of a flexible culture and the levers of control system: the key role of beliefs control. Manag Account Res 33:25–41

[CR48] Hipp L, Bünning M, Munnes S, Sauermann A (2020) Problems and pitfalls of retrospective survey questions in COVID-19 studies. Surv Res Methods 14(2):109–114

[CR49] Illegems V, Verbeke A (2004) Telework: what does it mean for management? Long Range Plan 37(4):319–334

[CR50] Jaspers E, Lubbers M, De Graaf ND (2009) Measuring once twice: an evaluation of recalling attitudes in survey research. Eur Sociol Rev 25(3):287–301

[CR51] Jensen MC, Meckling WH (1976) Theory of the firm: managerial behavior, agency costs and ownership structure. J Financ Econ 3(4):305–360

[CR52] Kennedy FA, Widener SK (2019) Socialization mechanisms and goal congruence. Acc Organ Soc 76:32–49

[CR53] Ketokivi M, Castaner X (2004) Strategic planning as an integrative device. Adm Sci Q 49(3):337–365

[CR54] KfW (2021) Unternehmensbefragung 2021: Corona-Krise belastet Unternehmen – Finanzierungsklima trübt sich ein

[CR55] Kober R, Ng J, Paul BJ (2007) The interrelationship between management control mechanisms and strategy. Manag Account Res 18(4):425–452

[CR56] Kristensen TB, Israelsen P (2014) Performance effects of multiple control forms in a lean organization: a quantitative case study in a systems fit approach. Manag Account Res 25(1):45–62

[CR57] Lill JB (2020) When the Boss is far away and there is shared pay: The effect of monitoring distance and compensation interdependence on performance misreporting. Acc Organ Soc 86:101143

[CR58] Malmi T, Bedford DS, Brühl R, Dergård J, Hoozée S, Janschek O, Willert J, Ax C, Bednarek P, Gosselin M, Hanzlick M, Israelsen P, Johanson D, Johanson T, Madsen DØ, Rohde C, Sandelin M, Strömsten T, Toldbod T (2020) Culture and management control interdependence: an analysis of control choices that complement the delegation of authority in Western cultural regions. Acc Organ Soc 86:101116

[CR59] Martin BH, MacDonnell R (2012) Is telework effective for organizations?: A meta-analysis of empirical research on perceptions of telework and organizational outcomes. Manag Res Rev 35(7):602–616

[CR60] McKinsey (2020) A dual cybersecurity mindset for the next normal. McKinsey Insights

[CR61] Mehmetoglu M, Venturini S (2021) Structural equation modelling with partial least squares using stata and R, 1st edn. CRC Press

[CR62] Merchant K, van der Stede W (2017) Management control systems, 4th edn. Prentice Hall, Upper Saddle River

[CR63] Milgrom PR, Roberts JD (1992) Economics, organization and management. Pearson

[CR64] Nutt PC (1989) Selecting tactics to implement strategic plans. Strateg Manag J 10(2):145–161

[CR65] Otley DT (1980) The contingency theory of management accounting: achievement and prognosis. Acc Organ Soc 5(4):413–428

[CR66] Otley DT (1994) Management control in contemporary organizations: toward a wider framework. Manag Account Res 5(3–4):289–299

[CR67] Ouchi WG (1977) The relationship between organizational structure and organizational control. Administ Sci Quart:95–113

[CR68] Perez M, Sanchez AM, de Luis Carnicer MP (2002) Benefits and barriers of telework: perception differences of humanresources managers according to company’s operations strategy. Technovation 22:775–783

[CR69] Petter S, Straub D, Rai A (2007) Specifying formative constructs in information systems research. Manag Inf Syst Q 31(4):623–656

[CR70] Podsakoff PM, Organ DW (1986) Self-reports in organizational research: problems and prospects. J Manag 12:531–544

[CR71] Raghuram S, Wiesenfeld B (2004) Work-Nonwork conflict and job stress among virtual workers. Hum Resour Manage 43(2):259–277

[CR72] Roberts J (2004) The modern firm. Oxford Univ Press

[CR73] Sardeshmukh SR, Sharma D, Golden TD (2012) Impact of telework on exhaustion and job engagement: a job demands and job resources model. N Technol Work Employ 27(3):193–207

[CR74] Sarstedt M, Hair JF, Cheah JH, Becker JM, Ringle CM (2019) How to specify, estimate, and validate higher-order constructs in PLS-SEM. Australas Mark J 27(3):197–211

[CR75] Schröder, C., Goebel, J., Grabka, M., Graeber, D., Kroh, M., Kröger, H., Kühne, S., Liebig, S., Schnupp, J., Seebauer, J., and Zinn, S. (2020). Erwerbstätige sind vor dem Covid-19-Virus nicht alle gleich (No. 1080; SOEPpapers on Multidisciplinary Panel Data Research)

[CR76] Sewell G, Taskin L (2015) Out of sight, out of mind in a new world of work? Autonomy, control, and spatiotemporal scaling in telework. Organ Stud 36(11):1507–1529

[CR77] Simons R (1995) Levers of control: how managers use innovative control systems to drive strategic renewal. Harvard Business School Press, Boston

[CR78] Teevan J, Hecht B, Jaffe S (2020) The new future of work. Microsoft Internal Rep.

[CR79] Vogel D (2018) Matching survey responses with anonymity in environments with privacy concerns: a practical guide. Int J Publ Sect Manag

[CR81] Widener SK (2007) An empirical analysis of the levers of control framework. Acc Organ Soc 32(7–8):757–788

[CR82] Workman M (2005) Virtual team culture and the amplification of team boundary permeability on performance. Hum Resour Dev Q 16(4):435–458

[CR83] Yang, L, Holtz D, Jaffe S, Suri S, Sinha S, Weston, J, Joyce C, Shah N, Sherman K, Hecht B, Teevan J (2022) The effects of remote work on collaboration among information workers. Nat Hum Behav 6:43-54 10.1038/s41562-021-01196-4 34504299

